# Supplementary material for: An analysis of NHS 111 demand for primary care services: A retrospective cohort study
Source: PLoS One. 2024 Jul 1;19(7):e0300193. doi: 10.1371/journal.pone.0300193 (PMC11216596; doi:10.1371/journal.pone.0300193)
Supplement: S1 Table — (PDF) [file pone.0300193.s002.pdf]

S1 Table: NHS pathways primary care dispositions

The table below contains the NHS Pathways clinical decision support software (CDSS) clinical disposition codes and descriptions for primary care, that are used by NHS 111.

Disposition code	Description
Dx05	To Contact a Primary Care Service within 2 hours
Dx06	To Contact a Primary Care Service within 6 hours
Dx07	To Contact a Primary Care Service within 12 hours
Dx08	To Contact a Primary Care Service within 24 hours
Dx11	Speak to a Primary Care Service within 1 Hour
Dx12	Speak to a Primary Care Service within 2 hours
Dx13	Speak to a Primary Care Service within 6 hours
Dx14	Speak to a Primary Care Service within 12 hours
Dx15	Speak to a Primary Care Service within 24 hours
Dx61	Speak to the GP Practice within 20 minutes
Dx75	Must Contact own GP Practice within 3 Working days
