# Supplementary material for: An analysis of NHS 111 demand for primary care services: A retrospective cohort study
Source: PLoS One. 2024 Jul 1;19(7):e0300193. doi: 10.1371/journal.pone.0300193 (PMC11216596; doi:10.1371/journal.pone.0300193)
Supplement: S2 Table — (PDF) [file pone.0300193.s003.pdf]

S4 Table: Direct booking by 111 call handler

Only GP appointments appeared to be bookable by the 111 call handler based on the data in this cohort, although this was infrequently undertaken and mostly ‘in-hours’

	Direct booking made by 111		
**Characteristic**	**No**, N = 47,979	**Yes**, N = 6,037	**Overall**, N = 54,016
Time of index 111 call (N, %)			
In-hours	9,234 (19%)	4,726 (78%)	13,960
Out-of-hours	38,745 (81%)	1,311 (22%)	40,056
Triaged primary care contact timeframe (N, %)			
1hr	10,014 (21%)	219 (3.6%)	10,233
2hrs	18,340 (38%)	1,207 (20%)	19,547
6hrs	11,262 (23%)	1,262 (21%)	12,524
>6hrs	8,363 (17%)	3,349 (55%)	11,712
First service contacted following index 111 call (N, %)			
999	667 (1.4%)	46 (0.8%)	713
ED	3,236 (6.7%)	284 (4.7%)	3,520
GP	24,375 (51%)	1,828 (30%)	26,203
IP	420 (0.9%)	80 (1.3%)	500
IUC	2,164 (4.5%)	231 (3.8%)	2,395
No further healthcare service contact	17,117 (36%)	3,568 (59%)	20,685
