# Supplementary material for: An analysis of NHS 111 demand for primary care services: A retrospective cohort study
Source: PLoS One. 2024 Jul 1;19(7):e0300193. doi: 10.1371/journal.pone.0300193 (PMC11216596; doi:10.1371/journal.pone.0300193)
Supplement: S3 Table — (PDF) [file pone.0300193.s004.pdf]

S5 Table: Clinical advisor involvement in 111 calls

Clinicians (clinical advisors) are involved in calls in cases where the call handler cannot prioritise the main symptom, the caller response to three NHS Pathways questions is 'unsure', the caller has declared a pre-existing medical condition, refuses the allocated disposition or is deemed complex, and enquiries about medication or medical/surgical procedures. This happened infrequently in this cohort of callers.

	Clinical advisor involved in call		
**Characteristic**	**No**, N = 46,279	**Yes**, N = 9,823	**Overall**, N = 56,102
Time of index 111 call (N, %)			
Out-of-hours	33,667 (73%)	7,663 (78%)	41,330
In-hours	12,612 (27%)	2,160 (22%)	14,772
Triaged primary care contact timeframe (N, %)			
1hr	8,241 (18%)	2,107 (21%)	10,348
2hrs	16,850 (36%)	2,930 (30%)	19,780
6hrs	10,323 (22%)	2,571 (26%)	12,894
>6hrs	10,865 (23%)	2,215 (23%)	13,080
Triage symptom group (N, %)			
Other	26,600 (57%)	6,811 (69%)	33,411
Pain and/or Frequency Passing Urine	2,970 (6.4%)	284 (2.9%)	3,254
Unwell, Under 1 Year Old	2,273 (4.9%)	222 (2.3%)	2,495
Skin, Rash	1,999 (4.3%)	441 (4.5%)	2,440
Earache	2,216 (4.8%)	186 (1.9%)	2,402
Sore Throat or Hoarse Voice	1,952 (4.2%)	366 (3.7%)	2,318
Chest and Upper Back Pain	1,597 (3.5%)	511 (5.2%)	2,108
Vomiting	1,838 (4.0%)	218 (2.2%)	2,056
Lower Back Pain	1,716 (3.7%)	232 (2.4%)	1,948
Cough	1,579 (3.4%)	286 (2.9%)	1,865
Abdominal Pain	1,539 (3.3%)	250 (2.5%)	1,789
First service contacted following index 111 call (N, %)			
GP	22,178 (48%)	4,512 (46%)	26,690
No further healthcare contact	17,749 (38%)	4,000 (41%)	21,749
ED	3,258 (7.0%)	545 (5.5%)	3,803
IUC	2,100 (4.5%)	502 (5.1%)	2,602
999	567 (1.2%)	172 (1.8%)	739
IP	427 (0.9%)	92 (0.9%)	519
