# Supplementary figures and images for: An analysis of NHS 111 demand for primary care services: A retrospective cohort study
Source: PLoS One. 2024 Jul 1;19(7):e0300193. doi: 10.1371/journal.pone.0300193 (PMC11216596; doi:10.1371/journal.pone.0300193)

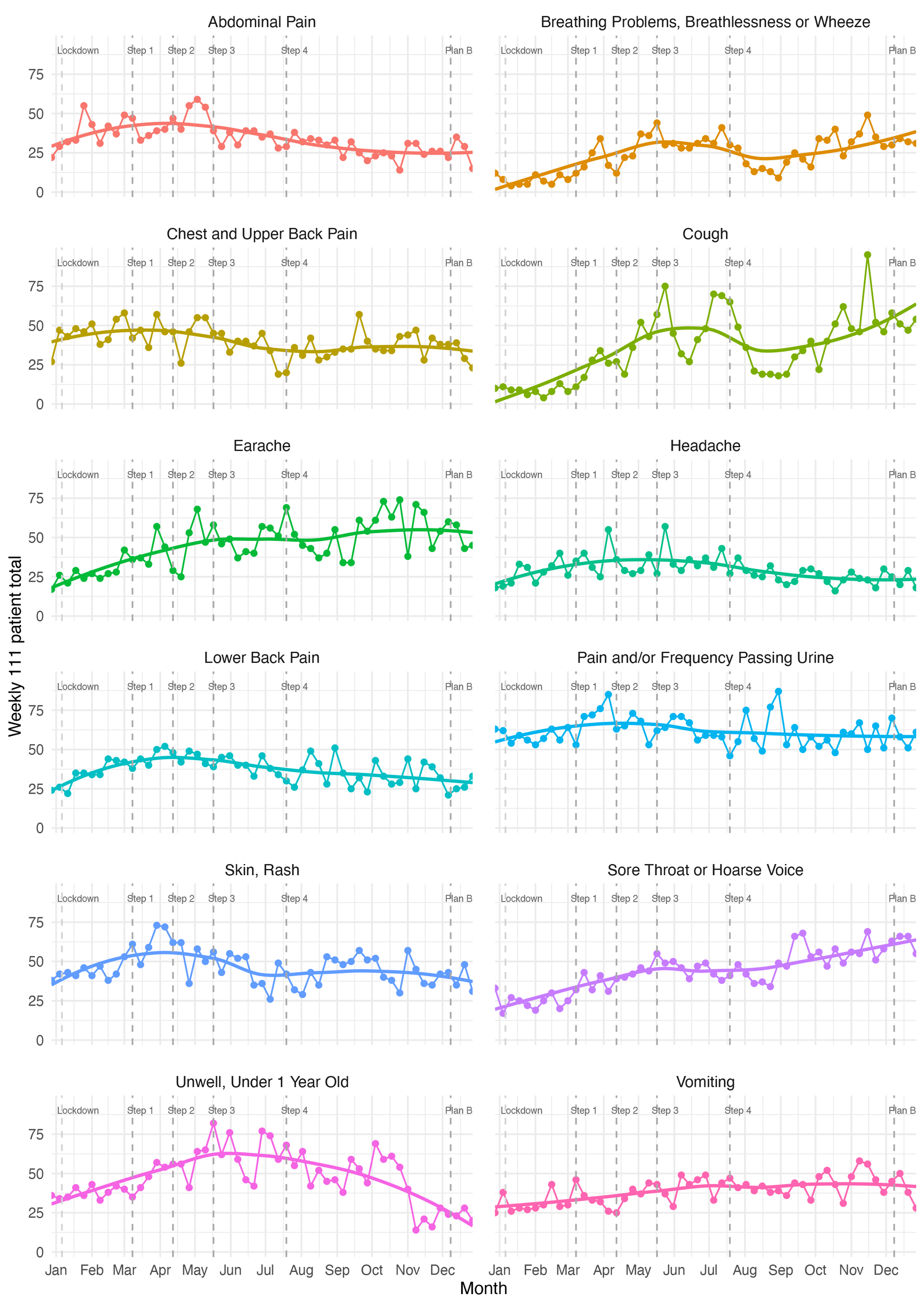

Supplement: S1 Fig — The study data collection period (January to December, 2021) coincided with the third English lockdown due to COVID-19. While several symptom group weekly frequencies did not change, for example pain on passing urine, others, particularly those which might be exacerbated by the relaxing of COVID-19 restrictions, for example coughs and sore throats, did see an increase. (TIF) [file pone.0300193.s005.tif]

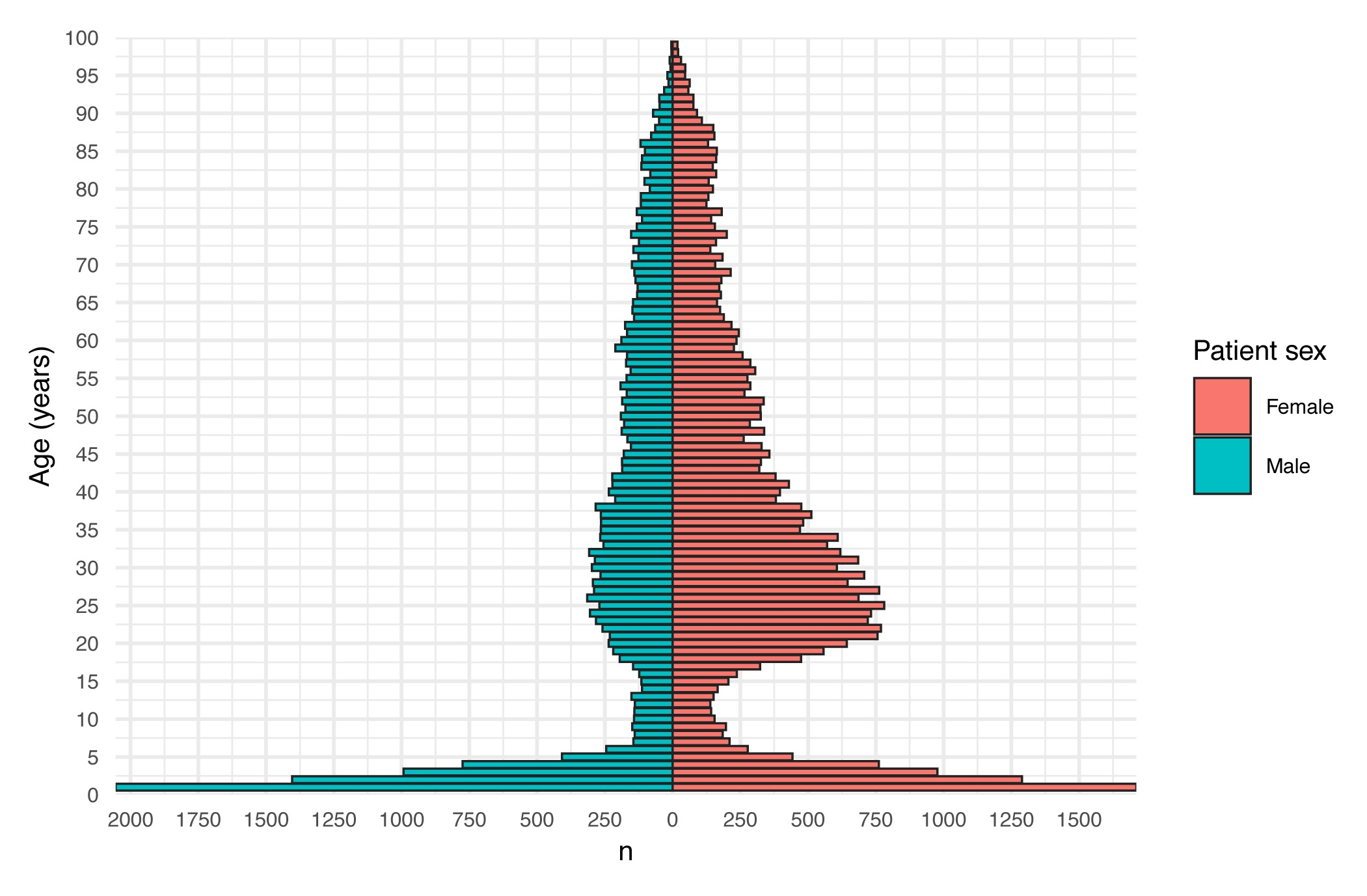

Supplement: S2 Fig — (TIF) [file pone.0300193.s006.tif]
