# Supplementary material for: An analysis of NHS 111 demand for primary care services: A retrospective cohort study
Source: PLoS One. 2024 Jul 1;19(7):e0300193. doi: 10.1371/journal.pone.0300193 (PMC11216596; doi:10.1371/journal.pone.0300193)
Supplement: Attachment — Submitted filename: MOOOD-paper1-resubmission-response-to-reviewers.docx [file pone.0300193.s007.docx]

Richard Pilbery
Yorkshire Ambulance Service NHS Trust Springhill, Brindley Way
Wakefield 41 Business Park, Wakefield. WF2 0XQ

Dr. Adam Aluisio

Academic Editor

PLOS ONE

Dear Dr. Aluisio and reviewers,

Thank you for your recent email regarding the outcome of the resubmission peer review. The only outstanding item is your request for an upload of the RECORD checklist, which I have completed.

In addition, there were a number of editorial administrative requests:

1. Manuscript meeting PLOS ONE style requirements. We have reviewed the PLOS ONE style requirements and are confident that our manuscript meets those outlined in the linked style documents.

2. Corresponding author affiliation. I have added my University of Sheffield affiliation, in addition to my clinical one.

3. Data availability statement. We have amended the statement to match the template suggested by PLOS One.

Reviewer’s comments

4. Previous publication. This is a poster abstract publication that was identified in an earlier submission. Following an appeal, where it was confirmed that I had followed the PLOS ONE guidance, this submission was reinstated.

Yours sincerely,

Richard Pilbery
